# Novel Lysophospholipid Acyltransferase PLAT1 of *Aurantiochytrium limacinum* F26-b Responsible for Generation of Palmitate-Docosahexaenoate-Phosphatidylcholine and Phosphatidylethanolamine

**DOI:** 10.1371/journal.pone.0102377

**Published:** 2014-08-04

**Authors:** Eriko Abe, Kazutaka Ikeda, Eri Nutahara, Masahiro Hayashi, Atsushi Yamashita, Ryo Taguchi, Kosaku Doi, Daiske Honda, Nozomu Okino, Makoto Ito

**Affiliations:** 1 Department of Bioscience and Biotechnology, Graduate School of Bioresource and Bioenvironmental Sciences, Kyushu University, Fukuoka, Japan; 2 Institute for Advanced Biosciences, Keio University, Kakuganji, Tsuruoka, Yamagata, Japan; 3 Faculty of Agriculture, University of Miyazaki, Miyazaki, Japan; 4 Faculty of Pharma-Sciences, Teikyo University, Tokyo, Japan; 5 Department of Biomedical Sciences, College of Life and Health Sciences, Chubu University, Kasugai-shi, Aichi, Japan; 6 Graduate School of Natural Science, Konan University, Higashinada-ku, Kobe, Hyogo, Japan; 7 Institute for Integrative Neurobiology, Konan University, Higashinada-ku, Kobe, Hyogo, Japan; 8 Faculty of Science and Engineering, Konan University, Higashinada-ku, Kobe, Hyogo, Japan; 9 Bio-Archtechture Center, Kyushu University, Fukuoka, Japan; Max Delbrueck Center for Molecular Medicine, Germany

## Abstract

N-3 polyunsaturated fatty acids (PUFA), such as docosahexaenoic acid (DHA, 22:6n-3), have been reported to play roles in preventing cardiovascular diseases. The major source of DHA is fish oils but a recent increase in the global demand of DHA and decrease in fish stocks require a substitute. Thraustochytrids, unicellular marine protists belonging to the Chromista kingdom, can synthesize large amounts of DHA, and, thus, are expected to be an alternative to fish oils. DHA is found in the acyl chain(s) of phospholipids as well as triacylglycerols in thraustochytrids; however, how thraustochytrids incorporate DHA into phospholipids remains unknown. We report here a novel lysophospholipid acyltransferase (PLAT1), which is responsible for the generation of DHA-containing phosphatidylcholine and phosphatidylethanolamine in thraustochytrids. The PLAT1 gene, which was isolated from the genomic DNA of *Aurantiochytrium limacinum* F26-b, was expressed in *Saccharomyces cerevisiae*, and the FLAG-tagged recombinant enzyme was characterized after purification with anti-FLAG affinity gel. PLAT1 shows wide specificity for donor substrates as well as acceptor substrates *in vitro*, *i.e*, the enzyme can adopt lysophosphatidylcholine, lysophosphatidylethanolamine, lysophosphatidylserine and lysophosphatidylinositol as acceptor substrates, and 15:0/16:0-CoA and DHA-CoA as donor substrates. In contrast to the *in vitro* experiment, only lysophosphatidylcholine acyltransferase and lysophosphatidylethanolamine acyltransferase activities were decreased in *plat1*-knockout mutants, resulting in a decrease of 16:0-DHA-phosphatidylcholine (PC) [PC(38∶6)] and 16:0-DHA-phosphatidylethanolamine (PE) [PE(38∶6)], which are two major DHA-containing phospholipids in *A. limacinum* F26-b. However, the amounts of other phospholipid species including DHA-DHA-PC [PC(44∶12)] and DHA-DHA-PE [PE(44∶12)] were almost the same in plat-knockout mutants and the wild-type. These results indicate that PLAT1 is the enzyme responsible for the generation of 16:0-DHA-PC and 16:0-DHA-PE in the thraustochytrid.

## Introduction

N-3 polyunsaturated fatty acids (n-3PUFA), such as docosahexaenoic acid (DHA, 22:6n-3) and eicosapentaenoic acid (EPA, 20:5n-3), have been reported to play roles in preventing cardiovascular diseases [1], [2]. DHA is the most abundant PUFA in the retina and brain and known to be necessary for their normal development/maturation in mammals, especially infants and children [3]–[5]. DHA is endogenously converted to resolvin D1, and protectin D1 by the actions of 12/15-lipoxygenase. These autacoids were shown to exert strong pro-resolving and anti-inflammatory actions [6]–[8].

Studies on the benefit of n-3 PUFA to human health have led to the use of fish oils in supplements and medicine; however, a recent increase in the global demand of n-3 PUFA and decrease in fish stocks mean that an alternative source to fish oils is needed [9]. Furthermore, it was reported that dietary n-3 PUFAs administered in phospholipid (PL) form were superior to those in triacylglycerol form for maintaining a healthy metabolic profile [10]. Fish oils include n-3 PUFA-containing triacylglycerols but not PLs, and, thus, a source of n-3 PUFA-containing PLs is required.

Although several lines of evidence have indicated the biological significance of DHA, the metabolism and functions of DHA-containing PLs *in vivo* remains unknown. One reason for lack of understanding of these mechanisms has been attributed to the absence of suitable model organisms for the synthesis of DHA and DHA-containing PLs. Although yeasts and *Escherichia coli* have greatly contributed to the accumulation of knowledge on lipid metabolism, neither can produce DHA. Thus, alternative model organisms that can produce DHA are strongly needed to further investigate the metabolism and functions of DHA and DHA-containing PLs.

Thraustochytrids, which are unicellular marine heterotrophic protists belonging to the Chromista kingdom, can produce large amounts of n-3 PUFAs, and, thus, are expected to be an alternative to fish oils [11], [12]. We have elucidated the structure of DHA-containing PLs in thraustochytrids [13], developed methods for thraustochytrid gene manipulation [14], [15], and revealed their n-3 PUFA synthesis pathways [16], [17]; however, how thraustochytrids synthesize DHA-containing PLs has yet to be elucidated.

In the present study, we identified a new lysophospholipid (LPL) acyl transferase (LPLAT) in the thraustochytrid, *Aurantiochytrium limacinum* F26-b, and named **PL** **A**cyl**T**ransferase 1 (PLAT1). PLAT1 shows wide specificity for donor substrates as well as acceptor substrates *in vitro*, *i.e*, the enzyme can adopt lysophosphatidylcholine (LPC), lysophosphatidylethanolamine (LPE), lysophosphatidylserine (LPS), and lysophosaphtidylinositol (LPI) as acceptor substrates and 15:0/16:0-CoA and DHA-CoA as donor substrates. However, disruption of the PLAT1 gene (*plat1*) in *A. limacinum* F26-b resulted in a decrease in 16:0-DHA-PC [PC(38∶6)] and 16:0-DHA-PE [PE(38∶6)], but not other PLs including DHA-DHA-PC [PC(44∶12)] and DHA-DHA-PE [PE(44∶12)]. This indicates that PLAT1 is the enzyme responsible for the generation of 16:0-DHA-PC and 16:0-DHA-PE, which are major PL species in the thraustochytrid. This is the first report on the identification of LPLAT in thraustochytrids, and the results obtained provide an insight into DHA metabolism in thraustochytrids, which are promising industrial microorganisms for the production of DHA and DHA-containing PLs. This study also suggests that *A. limacinum* F26-b is a favorable model organism for the metabolism and functions of DHA and DHA-containing PLs.

## Materials and Methods

### Materials

All acyl-CoAs were purchased from Avanti Polar Lipids (Alabaster, AL). [1-^14^C]palmitoyl-LPC (55 mCi/mmol) was obtained from Perkin-Elmer Life Sciences (Walthama, MA). Synthetic complete medium and the yeast nitrogen base were obtained from MP Biomedica (Morgan Irvine, CA). The yeast overexpression vector pYES2/CT and *S. cerevisiae* InvSc2 were purchased from Life Technologies Japan Ltd. (Tokyo, Japan). All other chemicals were obtained from either Sigma (St. Louis, MO) or Wako (Osaka, Japan). The sequences of primers used in this study are listed in Table S1.

### Identification of strain F26-b

The sequences were aligned with multiple-alignment method by using CLUSTAL_X [18] and refined manually. The alignment consists of 1,902 base pairs including gaps finally. The phylogenetic analyses were performed using MEGA 5.2 [19]. A neighbor-joining (NJ) phylogenetic tree was constructed using the distance matrix on the basis of the Tamura-Nei model [20]. Pairwise deletion was used for the gap treatment, and the statistical robustness of each branch was tested by a bootstrap analysis with 1,000 replications.

### Culture of strain F26-b

*A. limacinum* F26-b was grown in GY medium (3% glucose and 1% yeast extract in 50% artificial sea water) with or without 0.1% vitamin mixture (vitamin B_1_ 200 mg, vitamin B_2_ 1 mg, vitamin B_12_ 1 mg/100 ml distilled water) at 25°C for the period indicated. Cells were harvested by centrifugation at 3,000 rpm for 5 min. Potato dextrose agar (PDA) plates containing hygromycin (50% potato dextrose and 2% agar in 50% artificial sea water containing 2 mg/ml hygromycin) were used to select *plat1*-knockout mutants.

### Cloning of *plat1* from *A. limacinum* F26-b

LPLATs were searched for the genome database of *A. limacinum* ATCC MYA-1381 (http://genome.jgi.doe.gov/pages/blast.jsf?db=Aurli1) using known LPLAT sequences as a query. One of the hits, scaffold_4:802352-804647, showed an open reading frame (ORF) homologous to that of human LPCAT1 and contained four motifs conserved for LPLATs. We then picked up the sequence as a putative LPLAT gene of thraustochytrids.

The putative ORF was obtained from the genomic DNA from *A. limacinum* F26-b by PCR using primers 1 and 2 (Table S1). The amplified 1,938-base pair PCR product was cloned into the TA cloning vector pGEM-T Easy vector system (Promega). The insert was then sequenced using the BigDye Terminator v3.1 Cycle Sequencing Kit (Applied Biosystems) and 3130 Genetic Analyzer (Applied Biosystems). The gene and predicted protein were named *plat1* and PLAT1, respectively. The transmembrane motif was determined using the software TMHMM. A phylogenetic tree was drawn based on pairwise comparisons of the amino acid sequences of AGPAT (LPAAT), diacylglycerol acyltransferase 2 (DGAT2), and MBOAT family members using CLUSTALW, the DNA Data Bank of Japan (DDBJ) (http://clustalw.ddbj.nig.ac.jp/top-j.html).

### Expression of PLAT1 in *S. cerevisiae*

To construct the *plat1* expression vector, primers were designed to amplify the *plat1* ORF. The FLAG epitope (DYKDDDDK) was attached to the *N* terminus of PLAT1 by PCR using primers 3 and 4 (Table S1). The fragments were then released by *EcoR*I and *Not*I double digestion and inserted into the yeast expression vector pYES2/CT, which is designed to work under the control of the inducible promoter GAL1.

The yeast strain InvSc2 was transformed with the expression construct using the lithium acetate method, and transformants were selected on minimal medium lacking uracil. Transformants were first grown on minimal medium containing 2% D-glucose, and then cultured on minimal medium containing 2% D-galactose at 30°C.

### Preparation of yeast cell lysates

After the expression of *plat1* was induced with D-galactose, cells were harvested, re-suspended in 1 ml of ice-cold 20 mM Tris-HCl, pH 7.5, containing 240 mM sucrose and 0.2 M PMSF, and crushed with 0.5 mm glass beads by a BEAD BEATER (Biospec Inc.) 5 times for 30 s. After centrifugation at 3,000 rpm for 10 min, the supernatant was subjected to further centrifugation at 100,000×g for 1 h by an ultracentrifuge. The supernatant and pellet were collected separately as cytosolic and microsomal fractions, respectively. The protein concentration of each fraction was measured with a BCA assay kit using bovine serum albumin as a standard.

### Western blot analysis

Ten micrograms of total cellular protein was separated by 10% SDS-PAGE and transferred to a Hybond P nitrocellulose membrane (GE Healthcare). The membrane was blocked with 5% skim milk in TBS, incubated with the M2 anti-FLAG mouse monoclonal antibody in TBS, washed with TBS, and incubated with horseradish peroxidase (HRP)-linked anti-mouse IgG (GE Healthcare) in TBS. After being washed, the membranes were immersed in the peroxidase stain kit solution (Nacalai Tesque Inc.).

### Purification of PLAT1 by immunoprecipitation

FLAG-tagged PLAT1 (FLAG-PLAT1) was purified using the ANTI-FLAG M2 Affinity Gel and 3X FLAG peptide (Sigma Aldrich, St. Louis, MO) according to the manufacturer's instructions. Briefly, 200 µg of the yeast cell lysate, which did or did not contain FLAG-PLAT1 (mock transfectant), was diluted in 20 mM Tris-HCl, pH 7.5, containing 0.2 M PMSF (wash buffer). This sample was subjected to FLAG resin, kept at 4°C overnight, and the supernatant was removed. The resin was washed three times with 0.5 ml wash buffer, and suspended in 100 µl of wash buffer.

### Assay for LPLAT activity

LPLAT activity was measured based on the transfer of fatty acid from fatty acyl-CoA to lysoPLs (LPLs). To measure LPCAT activity, various fatty acyl-CoAs were incubated with [1-^14^C]palmitoyl-LPC and the [^14^C]PC that formed was quantified. The reaction mixture contained 1 mM EDTA, 0.01% sodium cholate, 5 µM [1-^14^C]palmitoyl LPC (50,000 dpm/nmol), 25 µM of the respective acyl-CoA, and 10 µg of cell protein (cell lysate) or 10 µl of purified FLAG-PLAT1 in 100 µl of 100 mM Tris-HCl, pH 7.5. When the Ca^2+^ dependency of the enzyme was measured, EDTA was replaced with 1 mM EGTA or 1 mM CaCl_2_. The reaction mixture was incubated at 30°C for 20 min and the reaction was then stopped by adding CHCl_3_/CH_3_OH (2∶1, v/v). Total lipids were extracted and applied to a thin layer chromatography (TLC) plate, which was developed with CHCl_3_/CH_3_OH/H_2_O (65/25/4, v/v/v). The radioactivity of the corresponding bands was quantified using a FLA 5100 Bio-imaging analyzer (GE Healthcare). Other LPLAT activities were measured using the method described for the LPCAT assay except that [1-^14^C]palmitoyl-CoA and a non-radioactive acceptor (LPA, LPS, LPE, or LPI) were used instead of non-labeled fatty acyl CoA and [1-^14^C]palmitoyl LPC, respectively. Each assay contained 25 µM of LPL, 1 mM EDTA, 0.01% sodium cholate, 5 µM [1-^14^C]palmitoyl-CoA (50,000 dpm/nmol), and 10 µlof purified FLAG-PLAT1 in 100 µl of 100 mM Tris-HCl, pH 7.5.

### Construction of the *plat1* knockout vector

The promoter region of the *Thraustochytrium aureum* ubiquitin gene was used to drive the expression of a selection marker, the hygromycin- resistant gene, as described in [16]. The terminator region of the SV40 virus coat protein gene was used to terminate gene transcription. The linear gene construct, which contained the promoter, the hygromycin resistant gene, and terminator, is referred to hereafter as the hygromycin-resistant cassette (Figure S1A, B).

Primers 5 and 6 (Table S1) were designed to insert the hygromycin-resistant cassette into *plat1* ORF by homologous recombination, by which *Bgl*II and *Sal*I sites were added to the middle of the coding region. Amplified PCR products were digested and linearized with *Bgl*II and *Sal*I. The linear fragment was treated with *E. coli* alkaline phosphatase (BAP) (TOYOBO Inc.) to avoid self-ligation before ligation to the hygromycin-resistant cassette, which was liberated from pT-HygR by digestion with *Bgl*II and *Sal*I. The newly constructed plasmid (pKO-PLAThygR) was used as a template for PCR to prepare the linear fragment. The linear *plat1*-KO targeting vector is illustrated in Figure S1A.

### Transformation of *A. limacinum* F26-b with the *plat1*-KO targeting vector

*A. limacinum* F26-b was grown in 3 ml of GY medium at 25°C for 3 days. Cells were collected by centrifugation at 3,000 rpm for 5 min, and washed with distilled water. Cell pellets were resuspended in Nucleofector solution (Lonza) to a final concentration of 5×10^6^ cells/100 µl. Five micrograms of the KO targeting vector was added to 100 µl of the cell suspension. The mixture was transferred into a 1-mm-gap cuvette. The cuvette was set on a GENE PULSER II (BioRad), and pulsed twice (0.75 kV, 50 µF, 50 Ω. The cells were added to 1 ml of fresh GY medium and incubated at 25°C overnight. Finally, all cells were transferred to hygromycin-containing PDA agar plates.

### Southern blot analysis

Wet cell pellets from 50 ml of the *A. limacinum* F26-b culture were washed with sterile water and suspended in 10 ml of lysis buffer (50 mM NaCl, 10 mM EDTA, and 0.5% SDS in 20 mM Tris-HCl pH 8.0) containing 0.2 mg/ml proteinase K. The suspension was incubated at 55°C overnight, 10 µl of RNase A (100 µg/ml) was then added, and the incubation continued at 37°C for 2 h. Ten µg of genomic DNA was digested overnight at 37°C with *Hin*dIII. The digested DNA was fractionated on a 1% agarose gel by electrophoresis and transferred to a Hybond-N+ nylon membrane (GE Healthcare).

The probe was synthesized using a DIG DNA Labeling Kit (Roche Applied Science) according to the manufacturer's instructions and primers 7 and 8 (Table S1). The membrane was incubated at 47°C with the probe and signals were detected with a DIG Nucleic Acid Detection Kit (Roche Applied Science).

### Nano ESI-MS analysis

Chip-based nanoESI-MS analysis was performed using 4000Q TRAP with a chip-based ionization source, TriVersa NanoMate (Advion BioSystems, Ithaca, NY, USA). The ion spray voltage was set at 1.25 kV, gas pressure at 0.3 pound per square inch (psi), and flow rates at 200 nL/min. The scan range was set at m/z 400–1100, de-clustering potential at 100 V, collision energies at 50–70 V, and resolutions at Q1 and Q3, “unit”. The mobile phase composition was chloroform/methanol containing 0.1% ammonium formate (1/2, v/v). Total lipids were directly subjected to flow injection and selectivity was analyzed by precursor ion scanning of PC and PE from individual parental molecular species [21]. d18:1/12:0-sphingomyelin was added to the sample as an internal standard.

## Results

### Identification of strain F26-b

**Figure 1:**
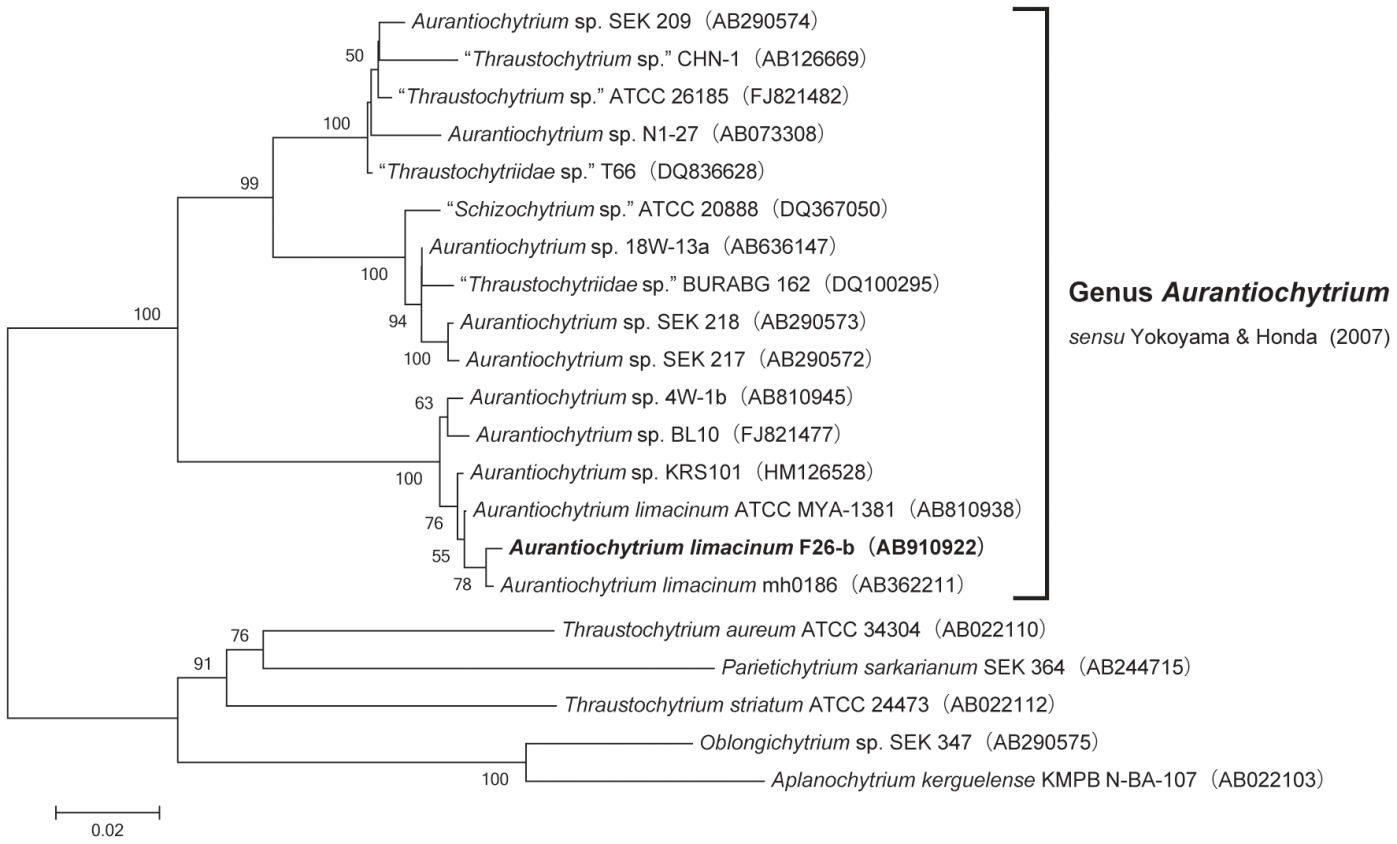
Identification of strain F26-b by 18S r RNA gene. A neighbor-joining tree was drawn based on 18S rRNA gene sequences of the genus *Aurantiochytrium* with five strains of other thraustochytrid genera as outgroups. Bootstrap values (>50%, 1,000 replicates) are shown on each internal branch. GenBank/DDBJ accession numbers of each sequence are indicated in parentheses.

The strain F26-b was isolated from fallen leaves of *Rhizophora mucronata* collected at Ishigaki Is., Okinawa, Japan. The neighbor-joining tree of 18S rRNA gene clearly shows that the strain F26-b forms a monophyletic group with the originally descripted ex-type strain (ATCC MYA 1381) of *A. limacinum* [22], [23] and four related strains that is strongly supported by the highest bootstrap value (100%) (Figure 1). The strain F26-b was identified as *A. limacinum* based on this phylogenetic position and the microscopic morphological features (not shown).

### Cloning of *plat1* from *A. limacinum* F26-b

**Figure 2:**
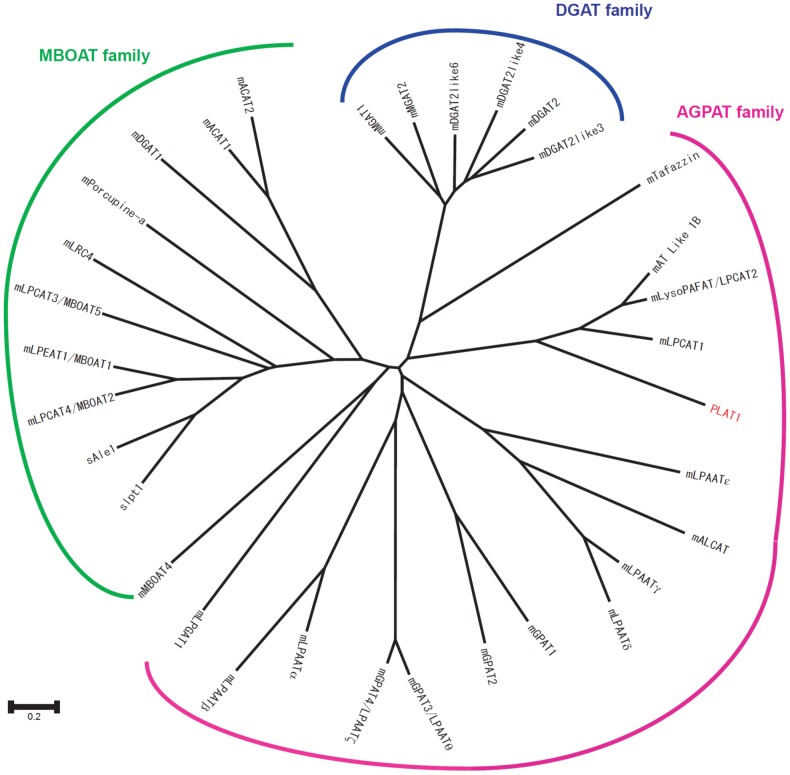
Alignment of PLAT1, mLPCAT1, and mLPCAT2. PLAT1 (this work), mLPCAT1 (LPCAT1 from mouse), and mLPCAT2 (LPCAT2 from mouse) sequences were aligned using GENETYX ver.8.2.2. The conserved amino acids are shown by white characters on a black background. The four conserved AGPAT motifs are indicated by red boxes. Two transmembrane regions, predicted by TMHMM server v. 2.0 (www.cbs.dtu.dk/services/TMHMM/), are underlined in blue. Three EF hand Ca^2+^-binding motifs, predicted by PROSITE (www.expasy.ch/prosite/), are indicated by green dashed-lines. ER-retaining motifs are indicated by red characters.

To identify the genes encoding LPLATs responsible for generating DHA-containing PLs in thraustochytrids, we searched the *A. limacinum* ATCC MYA-1381 genome database for sequence homology with previously reported LPLATs (Figure 2). We found seven candidate sequences in the database and designated them as PLAT1∼7, all of which belong to the AGPAT (LPAT) family. In this study, we cloned the PLAT1 gene (*plat1*) from the genomic DNA of *A. limacinum* F26-b, which is genealogically related to *A. limacinum* ATCC MYA-1381 (Figure 1).

**Figure 3:**
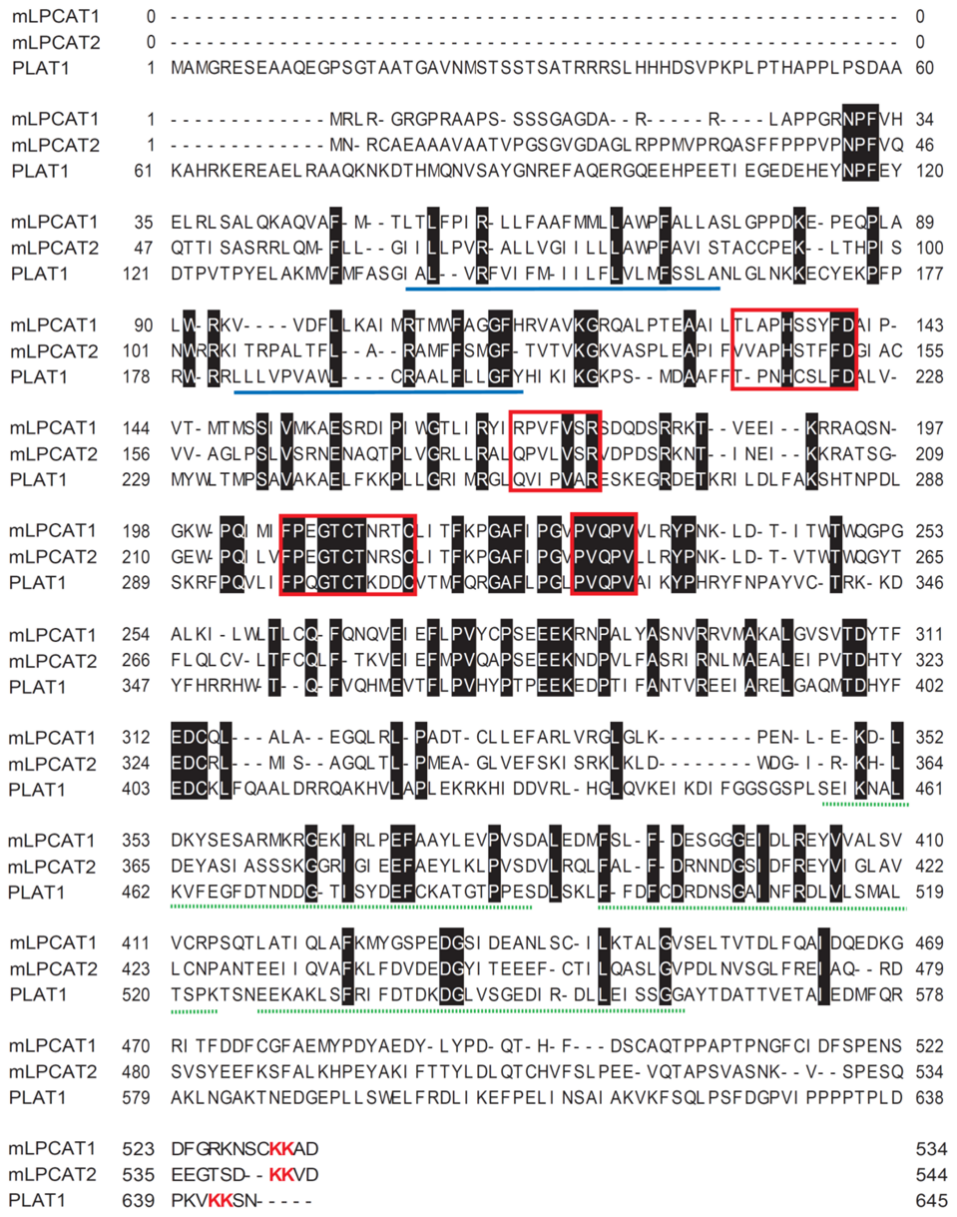
Phylogenetic tree of LPLAT family members. The phylogenetic tree was drawn using CLUSTALW, DDBJ (http://clustalw.ddbj.nig.ac.jp/top-j.html). LPLAT sequences are available on the NCBI database. The accession numbers are as follows: mGPAT1 (NP_032175), mGPAT2 (XP_130488), mGPAT3/LPAATθ (NP_766303), mLPAATα (NP_061350), mLPAATβ (NP_080488), mLPAATγ (NP_443747), mLPAATδ (NP_080920), mLPAATε (NP_081068), mGPAT4/LPAATζ (NP_061213), mAT Like 1B (NP_081875), mLPGAT1 (NP_758470), mALCAT (Q3UN02), mLPCAT1 (BAE94687), mLysoPAFAT/LPCAT2 (BAF47695), mTafazzin (NP_852657), mMGAT1 (NP_080989), mMGAT2 (NP_803231), mDGAT2 (NP_080660), mDGAT2Like3 (NP_001074605), mDGAT2Like4 (NP_808414), mDGAT2Like6 (CAM19588), mLPCAT3/MBOAT5 (NP_660112), mLPCAT4/MBOAT2 (NP_080313), mLPEAT1/MBOAT1 (NP_705774), mMBOAT4 (XP_134120), mDGAT1 (NP_034176), mACAT1 (NP_033256), mACAT2 (NP_666176), mPorcupine-a (NP_058609), mLRC4 (NP_084210), sLpt1 (BAF93897), and sAle1 (EWH15997); s, *Saccharomyces cerevisiae*, m, *Mus musculus*.

The putative ORF of *plat1* encoded a protein of 646 amino acid residues with a calculated molecular mass of 73.9 kDa. PLAT1 contained two transmembrane domains and four AGPAT motifs found in LPCATs [22], [25], [27]. Three EF hand motifs and a KK motif, which could serve as a calcium-binding motif and ER-retaining motif, respectively, were located at the C terminus (Figure 3). According to a phylogenetic tree based on the NCBI Blast search, PLAT1 was classified into the AGPAT (LPAAT) family, which includes mouse LPCAT1, LPCAT2 and GPATs with PLAT1 being the most homologous to mouse LPCAT1 (Figure 2).

### Characterization of PLAT1

**Figure 4:**
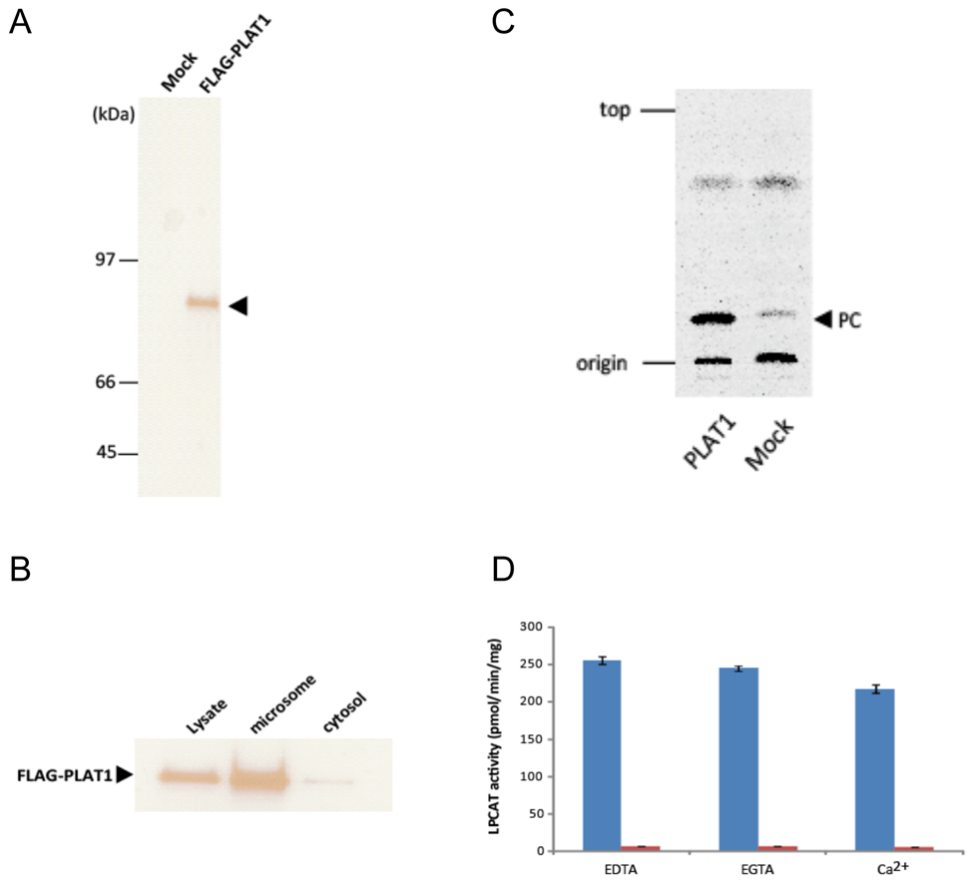
Western blot (A), intracellular localization (B), activity (C), and Ca^2+^-dependency (D) of FLAG-PLAT1 expressed in budding yeasts. (A) Western blot of FLAG-tagged PLAT1 at the N-terminal (FLAG-PLAT1). FLAG-PLAT1, expressed in yeast, was detected on 10% SDS-PAGE using the anti-FLAG mouse monoclonal antibody as the primary antibody, followed by HRP-conjugated anti-mouse IgG antibody as the secondary antibody. (B) The localization of PLAT1 was estimated by Western blot using the microsomal and cytosolic fractions of FLAG-PLAT1-expressing yeast cells. (C) LPCAT activity was measured based on the incorporation of 16∶0 from 16:0-CoA into [1-^14^C]palmitoyl-LPC. The reaction mixture contained 25 µM 16:0-CoA and 1 µM [1-^14^C]palmitoyl-LPC, and 10 µg of cell protein (cell lysate) in 100 µl of reaction buffer (100 mM Tris-HCl, pH 7.5, 1 mM EDTA, 0.01% sodium cholate). Mock represents the experiment using the enzyme prepared from the lysate of yeast cells harboring the empty vector. The reaction was conducted at 30°C for 20 min and terminated by the addition of 500 µl of CHCl_3_/CH_3_OH (2∶1, v/v). The reaction mixture was loaded on the TLC plate, developed with CHCl_3_/CH_3_OH/H_2_O (65/25/4, v/v/v), and analyzed with a FLA 5100 Bio-imaging analyzer. (D) LPCAT activity (left column) was measured by the method described in the legend (C), while the reaction mixture contained 1 mM EGTA (center column) or 1 mM CaCl_2_ (right column) instead of EDTA. The radioactivity of the band corresponding to PC on the TLC plate was quantified by a FLA 5100 Bio-imaging analyzer. Data represent the mean ± SD (n = 3). Blue and red bars represent the FLAG-PLAT1 and mock, respectively.

In order to characterize it, PLAT1 was expressed in *S. cerevisiae* as a fusion protein with a FLAG epitope at the N terminus. After the induction of PLAT1 expression by D-galactose, the yeast cell lysate was subjected to Western blotting using the M2 anti-FLAG antibody (Figure 4A). FLAG-PLAT1 showed a 74-kDa band on the blot consistent with the molecular mass predicted from the ORF of PLAT1. When the cell lysate was subjected to centrifugation to separate the cytosolic and microsomal fractions, FLAG-PLAT1 was mainly detected in the latter (Figure 4B). FLAG-PLAT1 possessed LPCAT activity when [^14^C]LPC and 16:0-CoA were used as an acceptor and donor, respectively (Figure 4C). Although PLAT1 had three EF hand calcium-binding motifs (Figure 3), Ca^2+^ was not necessary for LPCAT activity (Figure 4D).

**Figure 5:**
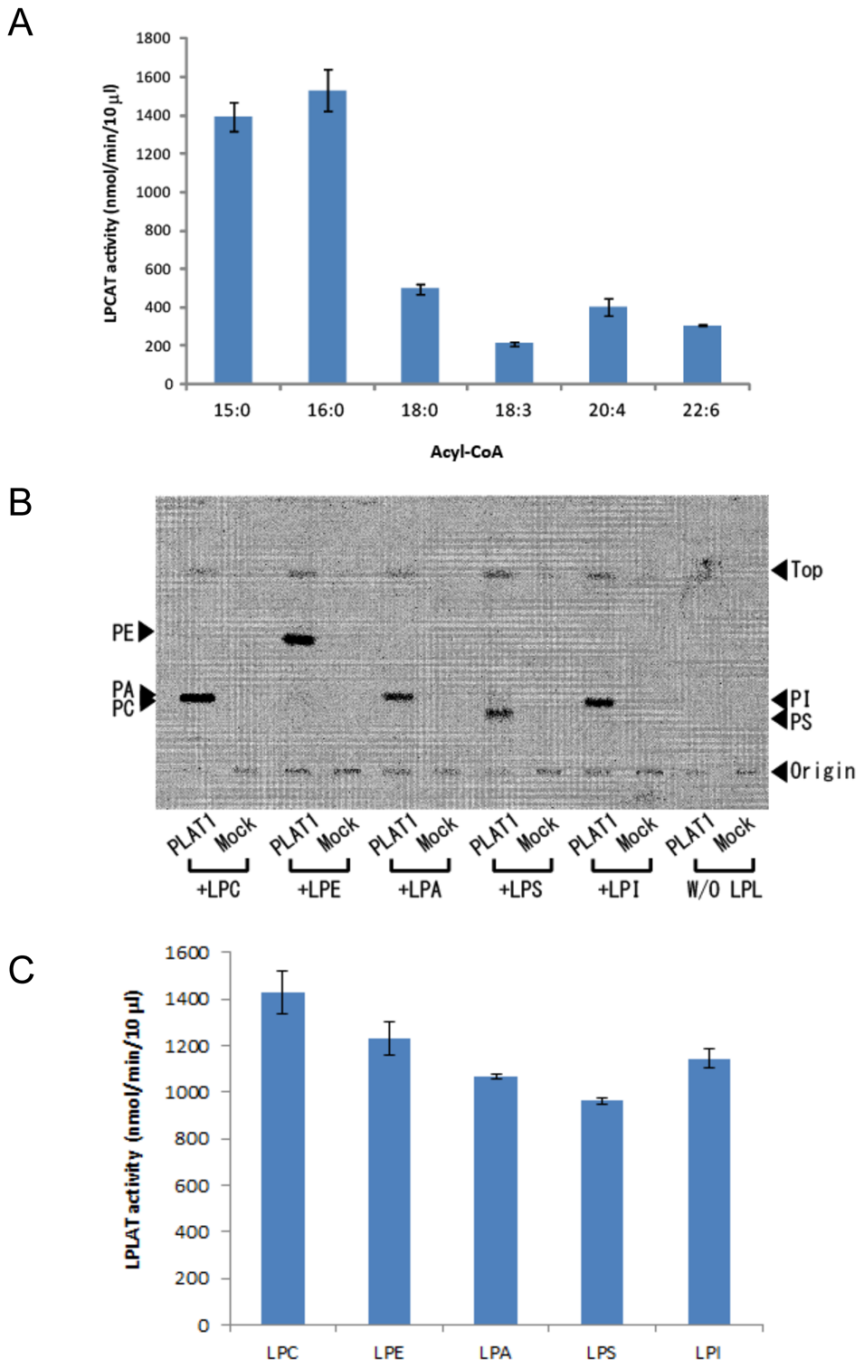
Specificity of PLAT1 for acyl-CoAs (A) and LPLs (B, C). (A) FLAG-PLAT1 was purified using the ANTI-FLAG M2 Affinity Gel and 3X FLAG peptide to remove the endogenous LPLs as described in Materials and Methods. Each assay contained 25 µM acyl-CoA, 1 µM [1-^14^C]palmitoyl-LPC, and purified enzyme (10 µl of gel suspension) in 100 µl of 100 mM Tris-HCl, pH 7.5, containing 1 mM EDTA and 0.01% sodium cholate. The reaction was conducted at 30°C for 20 min and terminated by the addition of 500 µl of CHCl_3_/CH_3_OH (2∶1, v/v). The reaction mixture was loaded on the TLC plate and developed with CHCl_3_/CH_3_OH/H_2_O (65/25/4, v/v/v). The radioactivity of the band corresponding to PC on the TLC plate was quantified by a FLA 5100 Bio-imaging analyzer. Data represent the mean ± SD (n = 3). (B) Each assay contained 1 µM of LPLs, 1 µM [1-^14^C]palmitoyl-CoA, and the purified enzyme (10 µl of gel suspension) in 100 µl of 100 mM Tris-HCl, pH 7.5, containing 1 mM EDTA and 0.01% sodium cholate. Mock represents the experiment using the enzyme prepared from the lysate of yeast cells harboring the empty vector. The assay was conducted at 30°C for 20 min and terminated by the addition of 500 µl of CHCl_3_/CH_3_OH (2∶1, v/v). The reaction mixture was loaded on the TLC plate, developed with CHCl_3_/CH_3_OH/H_2_O (65/25/4, v/v/v), and analyzed with a FLA 5100 Bio-imaging analyzer. The typical TLC is presented here. (C) The enzyme reaction was performed by the same procedure as shown in (B). The reaction products were applied on TLC plates and the radioactivity of the band corresponding to each PL was quantified by a FLA 5100 Bio-imaging analyzer (n = 3). Data represent the mean ± SD.

The specificity of PLAT1 toward acyl-CoA donors was examined using [^14^C]LPC and various fatty acyl-CoAs. To remove the endogenous LPLAT activity of *S. cerevisiae*, recombinant FLAG-PLAT1 was purified by immunoprecipitation using the anti-FLAG antibody immobilized on the agarose gel, as shown in the ‘Materials and Methods’. PLAT1 showed wide specificity and adopted various acyl-CoAs such as CoAs conjugated with 15∶0, 16∶0 (palmitic acid), 18∶0 (stearic acid), 18:3n-3 (α-linolenic acid), 20:4n-6 (arachidonic acid, AA), and 22:6n-3 (DHA). Although PLAT1 appeared to prefer 15:0-CoA and 16:0-CoA, it also adopted PUFA-CoAs such as 18:3-CoA, 20:4-CoA, and 22:6-CoA (Figure 5A). The specificity of PLAT1 toward acceptor substrates was examined using [^14^C]16:0-CoA and various LPLs. PLAT1 adopted LPC, LPE, LPA, LPI, and LPS as acceptor substrates, which indicated that the specificity of PLAT1 toward acceptor substrates was quite broad *in vitro* (Figure 5B, C). PLAT1 showed the relatively strong activity toward LPC among the acceptor substrates examined under the conditions used (Figure 5C).

### Generation and analysis of the *plat1*-disrupted mutants of *A. limacinum* F26-b

PLAT1 exhibited wide specificity for donors (Figure 5A) as well as acceptors (Figure 5B, C) *in vitro* when the FLAG-tag purified enzyme, expressed in *S. cerevisiae*, was used for the assay. To examine the *in vivo* specificity of PLAT1 in *A. limacinum* F26-b, *plat1* was disrupted in the thraustochytrid by homologous recombination using hygromycin as a selection marker (Figure S1A). Southern blotting showed that *plat1* was disrupted by the hygromycin-resistant gene (Figure S1B, C). The *plat1*-disrupted mutants of *A. limacinum* F26-b showed no obvious changes in cell growth and glucose consumption under the conditions used (Figure S2A, B).

**Figure 6:**
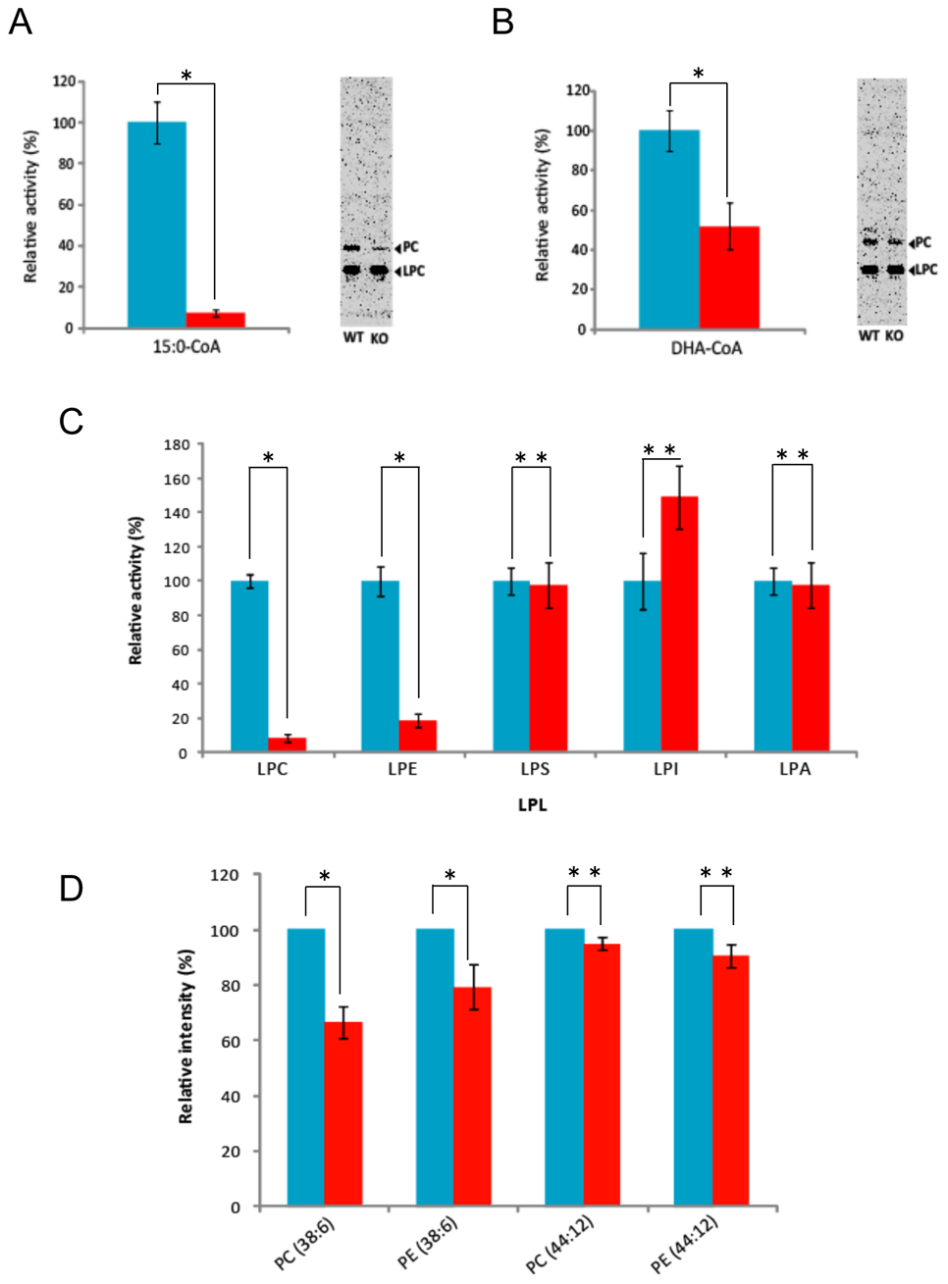
Disruption of the *plat1* gene in *A. limacinum* F26-b. (A, B) LPCAT activity was measured using 25 µM of 15:0-CoA (A) or DHA-CoA (B), 1 µM [1-^14^C]palmitoyl-LPC and 10 µg of the cell protein (cell lysate) from the *plat1*-knockout mutant (KO; red column) or wild-type (WT; blue column). (C) Various LPLAT activities were measured using 25 µM of the corresponding LPLs, 1 µM [1-^14^C]palmitoyl-CoA, 10 µg of the cell protein from KO (red column) or WT (blue column). Data represent the mean ± SD (n = 3). * and ** represent p<0.05 and not significant (p>0.10), respectively. (D) Change in the molecular species of PC and PE after the disruption of the *plat1* gene in *A. limacinum* F26-b. The PL fraction was prepared from *A. limacinum* F26-b before and after the disruption of the *plat1* gene. PLs were analyzed by Chip-based nanoESI-MS using a 4000Q TRAP with chip-based ionization source, TriVersa NanoMate (Advion BioSystems, Ithaca, NY, USA). The intensities at m/z 850 for 16:0-22:6-PC [PC(38∶6)], m/z 764 for 16:0-22:6-PE [PE(38∶6)], m/z 922 for 22:6-22:6-PC [PC(44∶12)], and m/z 836 for 22:6-22:6-PE [PE(44∶12)] were extracted and analyzed. The intensity of PL species of KO (red column) was expressed as a percentage of that of WT (blue column). Data represent the mean ± SD for 3 independent experiments (n = 1). * and ** represent p<0.05 and not significant (p>0.10), respectively.

A decrease in LPCAT activity was observed in *plat1*-disrupted mutants when activity was measured using LPC and 15:0-CoA or DHA-CoA as substrates; LPCAT activities were 90% and 80% lower than the wild-type when 15:0-CoA (Figure 6A) and 16:0-CoA (data not shown) were used, respectively, and 50% lower than the wild-type when DHA-CoA was used (Figure 6B). These results suggest that the acylation of LPC with saturated fatty acids was mainly catalyzed by PLAT1, while that with DHA was catalyzed by PLAT1 and other LPCATs in *A. limacinum* F26-b. The various LPLAT activities of *plat1*-disrupted mutants were examined using different LPLs and [^14^C]16:0-CoA. As shown in Figure 6C, the activities of not only LPCAT, but also LPEAT were significantly decreased after the disruption of the *plat1* gene; however, those of LPSAT and LPAAT were not, which indicated that PLAT1 is likely to function *in vivo* as LPCAT and LPEAT, but not other LPLATs. LPIAT activity was found to increase in *plat1*-knockout mutants under the conditions used (Figure 6C).

ESI-MS analysis revealed that the ratios of 16:0-DHA-PC [PC(38∶6)] and 16:0-DHA-PE [PE(38∶6)] were 35% and 20%, respectively, lower in *plat1*-knockout mutants than in the wild-type. However, the ratios of DHA-DHA-PC [PC(44∶12)] and DHA-DHA-PE [PE(44∶12)] were almost the same in *plat1*-knockout mutants as in the wild-type (Figure 6D). The amounts of other PL species were unchanged following the disruption of the *plat1* gene. The amounts of 16:0-DHA-PC and 16:0-DHA-PE were approximately 3∼4 times higher than those of DHA-DHA-PC and DHA-DHA-PE in the wild-type, and the ratio of 16:0-DHA-PC/PE to DHA-DHA-PC/PE decreased in the knockout mutants (Table S2). Neither 16:0-16:0-PC nor 16:0-16:0-PE was detected in *A. limacinum* F26-b under the conditions used. These results suggest that PLAT1 is responsible for the generation of 16:0-DHA-PC and 16:0-DHA-PE, which are major PLs in *A. limacinum* F26-b [13].

## Discussion

The major fatty acids of *A. limacinum* F26-b are 15:0/16:0 and DHA; the former occupies approximately 40% of total fatty acids, and the latter, approximately 30% when the strain was cultured in GY medium [13]. However, most of 15∶0 was replaced by palmitic acid (16∶0) with the addition of vitamin B12. The odd-chain fatty acid (15∶0) could also be generated from the fatty acid synthase (FAS) pathway using propionyl-CoA as a primer instead of malonyl-CoA in the absence of vitamin B12 [24], [25].

**Figure 7:**
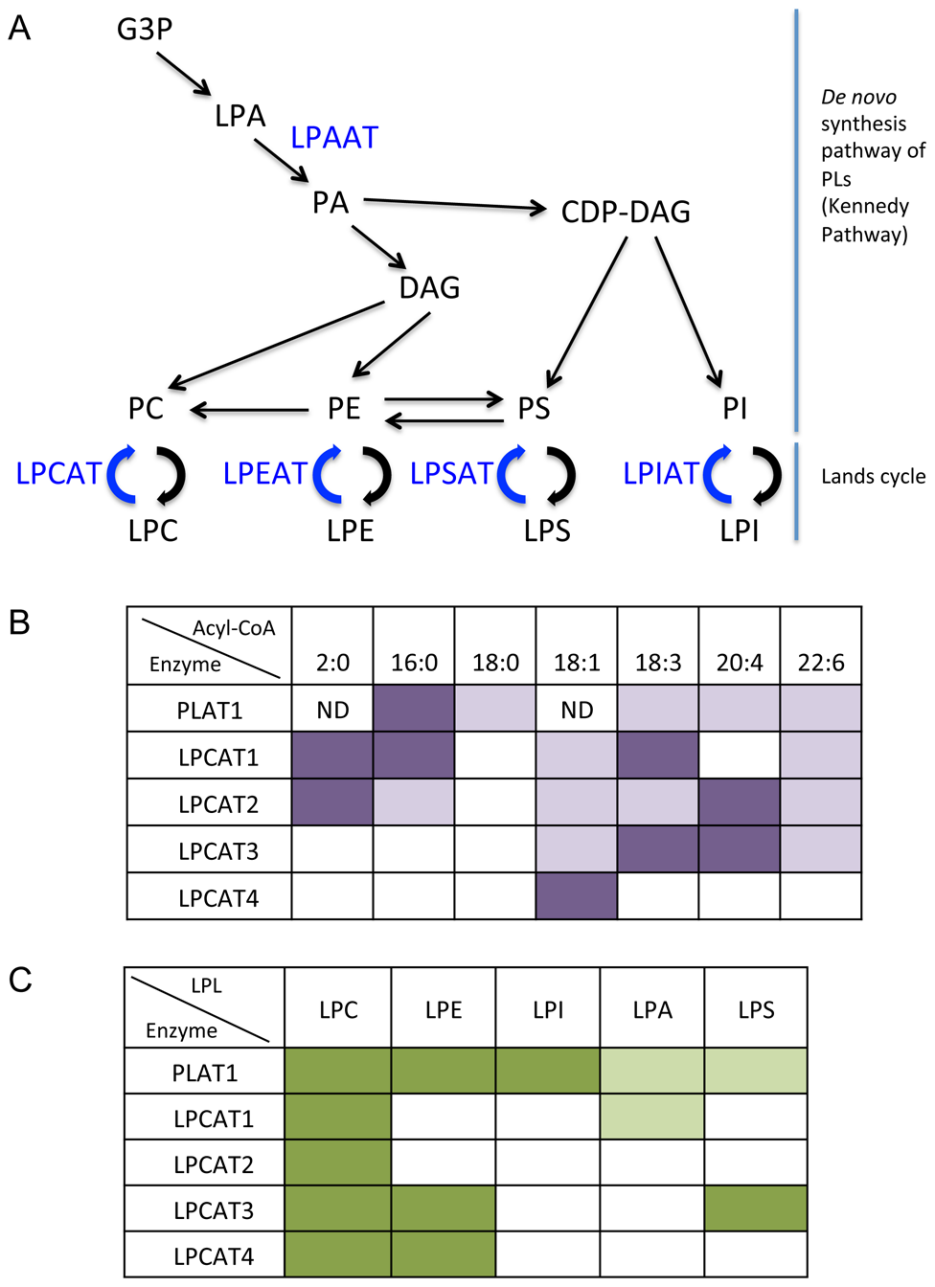
Metabolic map of PLs (A) and comparison of the specificity of PLAT1 with those of other LPCATs (B, C). Panel A represents the LPLATs shown in this study along with the metabolic map of PLs in mammals. The specificity of PLAT1 for acyl-CoAs (B) and LPLs (C) was compared with those of the LPCATs reported so far. The darker color represents higher activity toward the substrates indicated. White (blank) and ND show the absence of activity and not determined, respectively. The specificities of LPCATs are mainly referred from [27].

In general, PLs are synthesized from Kennedy pathway (Figure 7A) and the fatty acids at *sn*-2 are released from PLs through the actions of PLA_2_, and PLs are re-generated by transferring fatty acids from acyl-CoA to LPLs by LPLATs. This remodeling of the fatty acyl chains of PLs was proposed by Lands in the 1950s and has been named Lands cycle (Figure 7A) [26]. The LPLATs involved in Lands cycle were previously unknown, but may have recently been identified independently by several laboratories [27]–[32].

LPLATs are divided into two major groups based on their primary structures and characteristic motifs; AGPAT (LPAAT) and MBOAT (Figure 3) [33]–[35]. The enzymes belonging to AGPAT family have four well-conserved motifs [33]. The MBOAT family enzymes also possess the conserved motifs that are postulated to be important for the activity, although the motifs of MOBAT are totally different from those of AGPAT [36], [37]. Among the LPLATs reported to date, several enzymes were found to adopt PUFA-CoA as the donor substrate. LPIAT, which catalyzes the incorporation of arachidonic acid (AA, 20:4n-6) and EPA into LPI from the corresponding acyl donor, was isolated from *Caenorhabditis elegans* [38]. This was the first LPLAT that was shown to be capable of catalyzing the incorporation of PUFAs into PLs; however, *C. elegans* cannot synthesize DHA [39].

PLAT1, which is composed of 646 amino acid residues, possesses four conserved AGPAT motifs and three EF hand calcium-binding motifs. These structural features closely resemble those of mouse/human LPCAT1 and LPCAT2. When *plat1* was expressed in yeast cells, the LPCAT activity of recombinant PLAT1 was completely independent of Ca^2+^, similar to that of recombinant human LPCAT1 [30]. In contrast, LPCAT2 only exhibited lysoPAF acyltransferase activity in the presence of Ca^2+^ [32].

PLAT1 utilized both saturated and unsaturated fatty acyl-CoAs as acceptor substrates, similar to LPCAT1 and LPCAT2. Among the saturated fatty acyl-CoAs available, PLAT1 exhibited a preference for 15:0-CoA, 16:0-CoA, and 18:0-CoA, while LPCAT1 preferred 16:0-CoA, and LPCAT2, 2:0-CoA. In contrast to PLAT1, 18:0-CoA was not utilized by LPCAT1 or LPCAT2 [30]. Long chain PUFA-CoAs such as DHA-CoA and AA-CoA were utilized by PLAT1, whereas AA-CoA was utilized by LPCA2 but not LPCAT1. The specificities of LPCAT3 (MBOAT5) and LPCAT4 (MBOAT2), both belonging to the MBOAT family, were markedly different to that of PLAT1. LPCAT3 (MBOAT5) exhibited a preference for 18:2-CoA and AA-CoA but it did not utilize 16:0-CoA, 18:0-CoA, or DHA-CoA, whereas LPCAT4 appeared to be specific to 18:1-CoA [27].

The specificity of PLAT1 toward LPL acceptors *in vitro* was different to those of LPCAT1 and LPCAT2. Apart from LPCAT activity, PLAT1 also exhibited LPEAT, LPAAT, LPIAT, and LPSAT activities *in vitro*. In contrast, LPCAT1 exhibited LPAAT, LPGAT, and lysoPAF-AT activities, while LPCAT2 possessed a strong lysoPAF-AT activity in addition to primary LPCAT activity [32]. The comparison of the specificities of PLAT1 and other known LPCATs toward acyl-CoAs and LPLs is summarized in Figure 7 B, C.

To clarify the *in vivo* functions of PLAT1 in *A. limacinum* F26-b, *plat1*-knockout mutants were generated in this study. Recombinant PLAT1 showed the LPLAT activities toward not only LPC but also LPE, LPI, LPA and LPS under *in vitro* enzyme assay (Figure 5C). However, only LPCAT and LPEAT activities were significantly lower in the *plat1*-knockout mutants than in the wild-type (Figure 6C). This result may indicate that other LPLATs compensate the activities of LPIAT, LPAAT and LPSAT *in vivo*. Interestingly, nano ESI-MS analysis indicated that the amounts of 16:0-DHA-PC and 16:0-DHA-PE, but not DHA-DHA-PC and DHA-DHA-PE, were lower in *plat1*-knockout mutants than in the wild-type (Figure 6D). It is worth noting that neither 16:0-16:0-PC nor 16:0-16:0-PE was detected by nano ESI-MS in *A. limacinum* F26-b. These results indicate that PLAT1 may be central to the generation of 16:0-DHA-PC and 16:0-DHA-PE, which are major PL species in *A. limacinum* F26-b [13]. In this context, it is suggested that PLAT1 preferentially transfers 16:0 from 16:0-CoA to DHA-LPC (LPE); however, the enzyme could also adopt DHA-CoA as a donor substrate if the acceptor substrate is a 16:0-LPC (LPE) *in vivo*.

This study promotes understanding of the global lipid metabolism of thraustochytrids, and may accelerate the molecular breading of promising microorganisms for the production of DHA and DHA-containing phospholipids.

## Supporting Information

Figure S1 **Strategy for the disruption of the** ***plat1*** **gene in** ***A. limacinum*** **F26-b (A) and Southern blot analysis of the** ***plat1*** **-knockout mutants (B, C).** (A) The ORF of the *plat1* gene was disrupted by homologous recombination using a hygromycin expression cassette composed of a hygromycine-resistant gene (Hyg^r^) with an ubiquitin promoter and SV40 terminator. The *plat1* knockout cassette consisted of a Hyg^r^ expression cassette sandwiched between the 819-bp 5′- and 1077-bp 3′-flanking sequences of the *plat1* gene. (B) A map illustrating the location of the region annealing with the specific probe (red double arrows) and detectable restriction fragments obtained by HindIII digestion. (C) Southern blot of HindIII-restriction fragments from the wild-type (WT) and *plat1*-knockout mutant (KO) with a specific probe, as shown in (B).
(TIF)

Figure S2 **Growth (A) and glucose consumption (B) of the wild-type and** ***plat1*** **-disruption mutants of** ***A. limacinum*** **F26-b.** The wild-type and three *plat1*-disrupted mutants (*plat1*-KO1∼3) were cultured in 50-ml flasks containing 20 ml of GY medium (3% glucose and 1% yeast extract in 50% artificial sea water) containing 0.1% vitamin mixture (vitamin B_1_: 200 mg, vitamin B_2_: 1 mg, vitamin B_12_: 1 mg/100 ml distilled water) at 25°C for the periods indicated. A small sample of the culture was withdrawn and the optical density at 600 nm was measured after suitable dilution. The glucose content of the culture supernatant was measured with a glucose CII-test (Wako, Japan). Data represent the mean ± SD (n = 3).
(TIF)

Table S1 PCR primers used in this study. Underlines, restriction enzyme sites (No. 3 *Hin*dIII, No. 4 *Bam*HI, No. 5 *Bgl*II, No. 6 *Sal*I); Dotline, FLAG tag sequence.
(DOCX)

Table S2 Ratios of PL species in wild-type and *plat1*-disrupted mutants. Values are the ratios of 16:0-DHA-PLs to DHA-DHA-PLs, calculated from the data of nanoESI/MS analyses. WT, wild-type of *A.limacinum* F26-b; KO1∼KO3, three different *plat1*-disrupted mutants obtained from transfection of the wild-type with a KO construct containing the HygR gene as a marker, as shown in Figure S1.
(DOCX)
