# Correction: Novel Lysophospholipid Acyltransferase PLAT1 of *Aurantiochytrium limacinum* F26-b Responsible for Generation of Palmitate-Docosahexaenoate-Phosphatidylcholine and Phosphatidylethanolamine

**DOI:** 10.1371/journal.pone.0203016

**Published:** 2018-08-23

**Authors:** Eriko Abe, Kazutaka Ikeda, Eri Nutahara, Masahiro Hayashi, Atsushi Yamashita, Ryo Taguchi, Kosaku Doi, Daiske Honda, Nozomu Okino, Makoto Ito

The titles and legends for Figs 2 and 3 are swapped. Please see the correct Figs 2 and 3 here.

**Fig 2:**
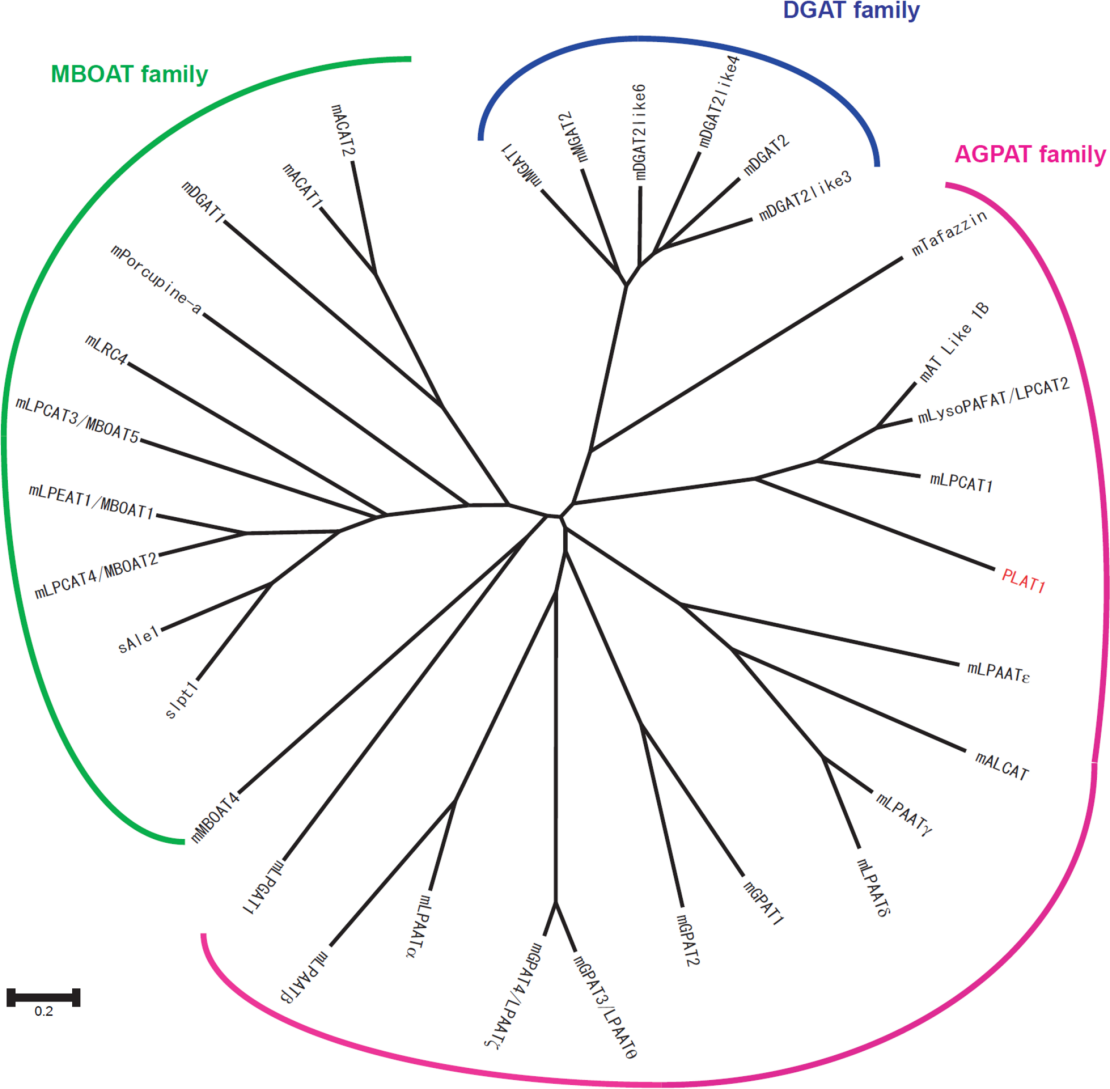
Phylogenetic tree o f LPLAT family members. The phylogenetic tree was drawn using CLUSTALW, DDBJ (http://clustalw.ddbj.nig.ac.jp/top-j.html). LPLAT sequences are available on the NCBI database. The accession numbers are as follows: mGPAT1 (NP_032175), mGPAT2 (XP_130488), mGPAT3/LPAATh (NP_766303), mLPAATa (NP_061350), mLPAATb (NP_080488), mLPAATc (NP_443747), mLPAATd (NP_080920), mLPAATe (NP_081068), mGPAT4/LPAATf (NP_061213), mAT Like 1B (NP_081875), mLPGAT1 (NP_758470), mALCAT (Q3UN02), mLPCAT1 (BAE94687), mLysoPAFAT/LPCAT2 (BAF47695), mTafazzin (NP_852657), mMGAT1 (NP_080989), mMGAT2 (NP_803231), mDGAT2 (NP_080660), mDGAT2Like3 (NP_001074605), mDGAT2Like4 (NP_808414), mDGAT2Like6 (CAM19588), mLPCAT3/MBOAT5 (NP_660112), mLPCAT4/MBOAT2 (NP_080313), mLPEAT1/MBOAT1 (NP_705774), mMBOAT4 (XP_134120), mDGAT1 (NP_034176), mACAT1 (NP_033256), mACAT2 (NP_666176), mPorcupine-a (NP_058609), mLRC4 (NP_084210), sLpt1 (BAF93897), and sAle1 (EWH15997); s, *Saccharomyces cerevisiae*, m, *Mus musculus*.

**Fig 3:**
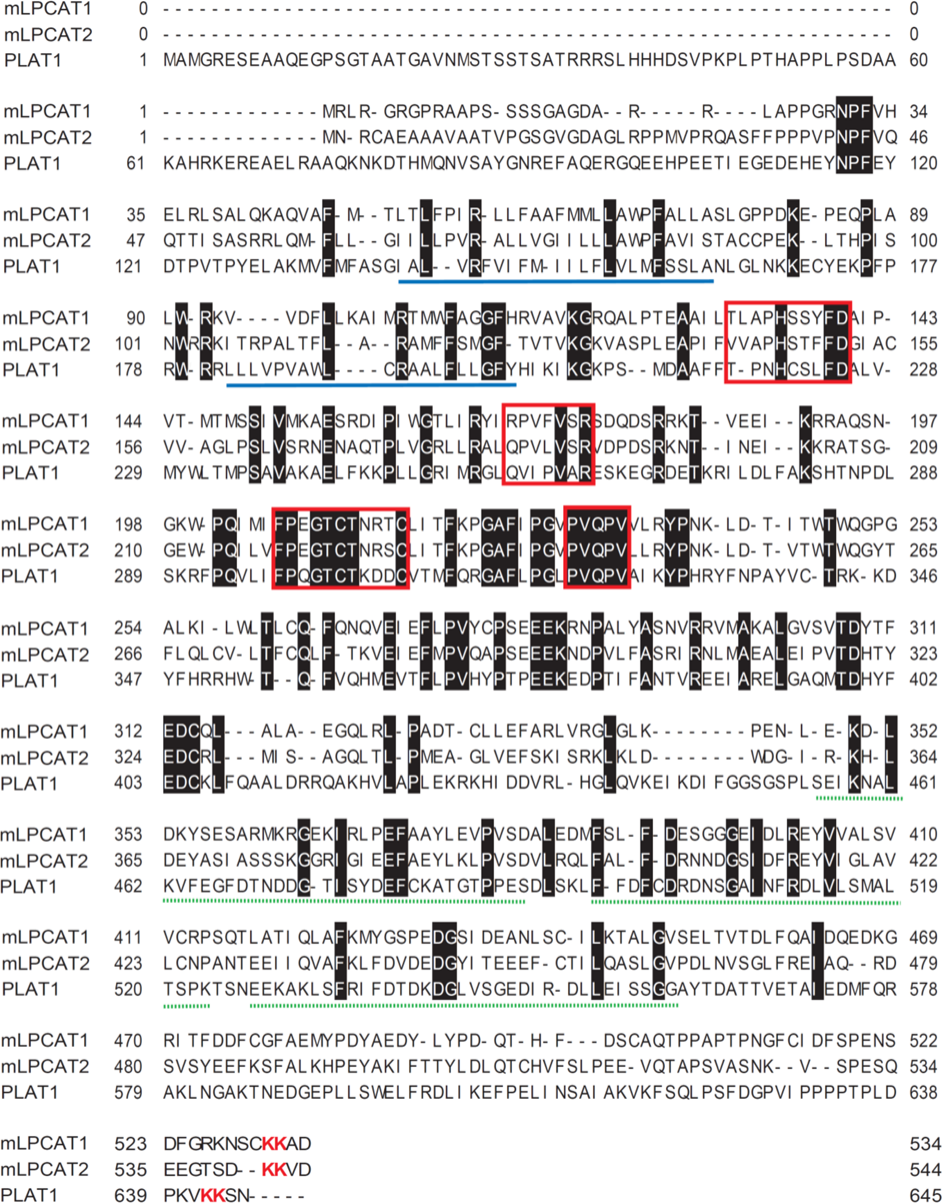
Alignment of PLAT1, mLPCAT1, and mLPCAT2. PLAT1 (this work), mLPCAT1 (LPCAT1 from mouse), and mLPCAT2 (LPCAT2 from mouse) sequences were aligned using GENETYX ver.8.2.2. The conserved amino acids are shown by white characters on a black background. The four conserved AGPAT motifs are indicated by red boxes. Two transmembrane regions, predicted by TMHMM server v. 2.0 (www.cbs.dtu.dk/services/TMHMM/), are underlined in blue. Three EF hand Ca^2+^-binding motifs, predicted by PROSITE (www.expasy.ch/prosite/), are indicated by green dashed-lines. ER-retaining motifs are indicated by red characters.
