# Supplementary figures and images for: Novel Lysophospholipid Acyltransferase PLAT1 of Aurantiochytrium limacinum F26-b Responsible for Generation of Palmitate-Docosahexaenoate-Phosphatidylcholine and Phosphatidylethanolamine
Source: PLoS One. 2014 Aug 4;9(8):e102377. doi: 10.1371/journal.pone.0102377 (PMC4121067; doi:10.1371/journal.pone.0102377)

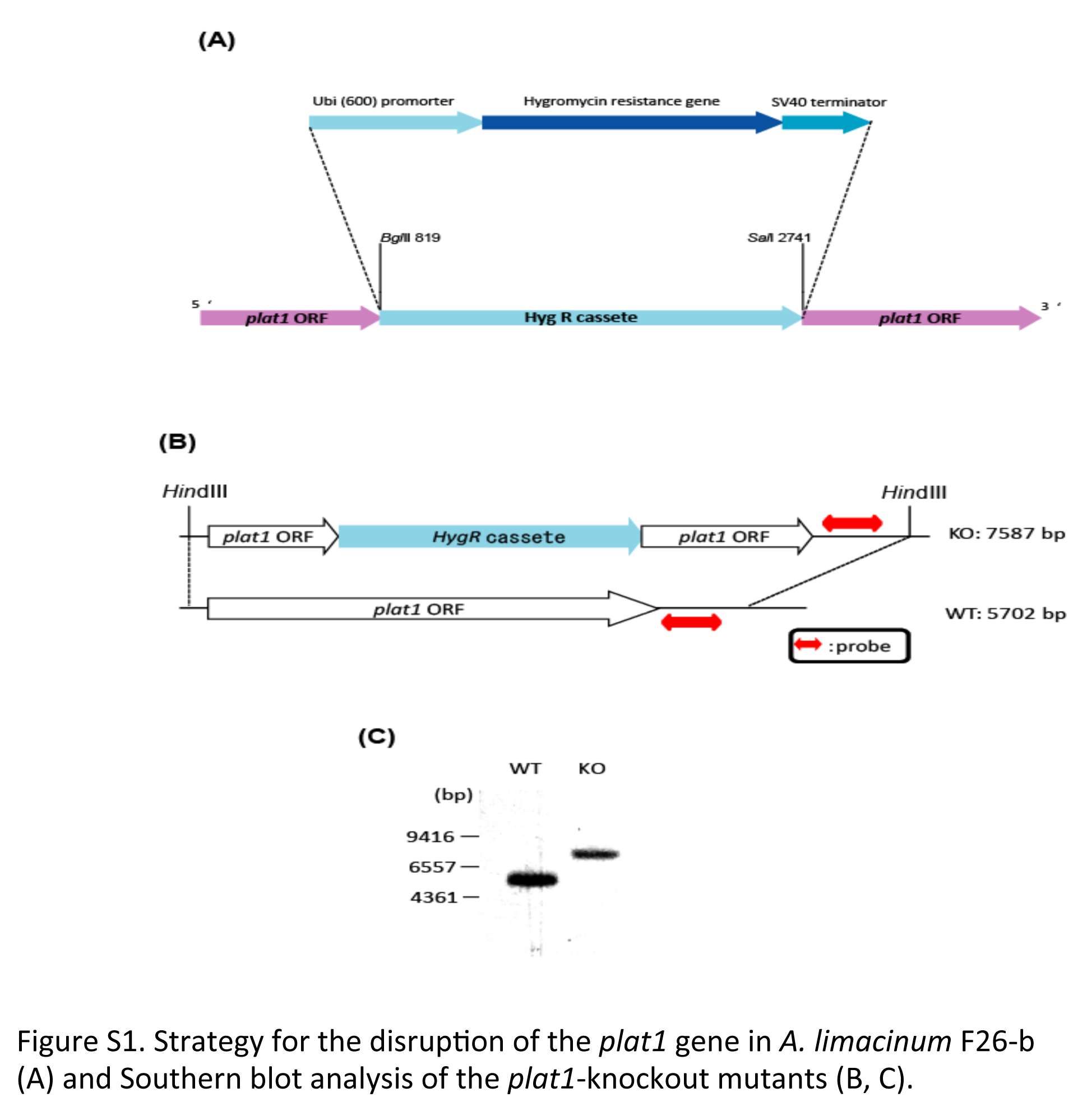

Supplement: Figure S1 — Strategy for the disruption of the plat1 gene in A. limacinum F26-b (A) and Southern blot analysis of the plat1 -knockout mutants (B, C). (A) The ORF of the plat1 gene was disrupted by homologous recombination using a hygromycin expression cassette composed of a hygromycine-resistant gene (Hygr) with an ubiquitin promoter and SV40 terminator. The plat1 knockout cassette consisted of a Hygr expression cassette sandwiched between the 819-bp 5′- and 1077-bp 3′-flanking sequences of the plat1 gene. (B) A map illustrating the location of the region annealing with the specific probe (red double arrows) and detectable restriction fragments obtained by HindIII digestion. (C) Southern blot of HindIII-restriction fragments from the wild-type (WT) and plat1-knockout mutant (KO) with a specific probe, as shown in (B). (TIF) [file pone.0102377.s001.tif]

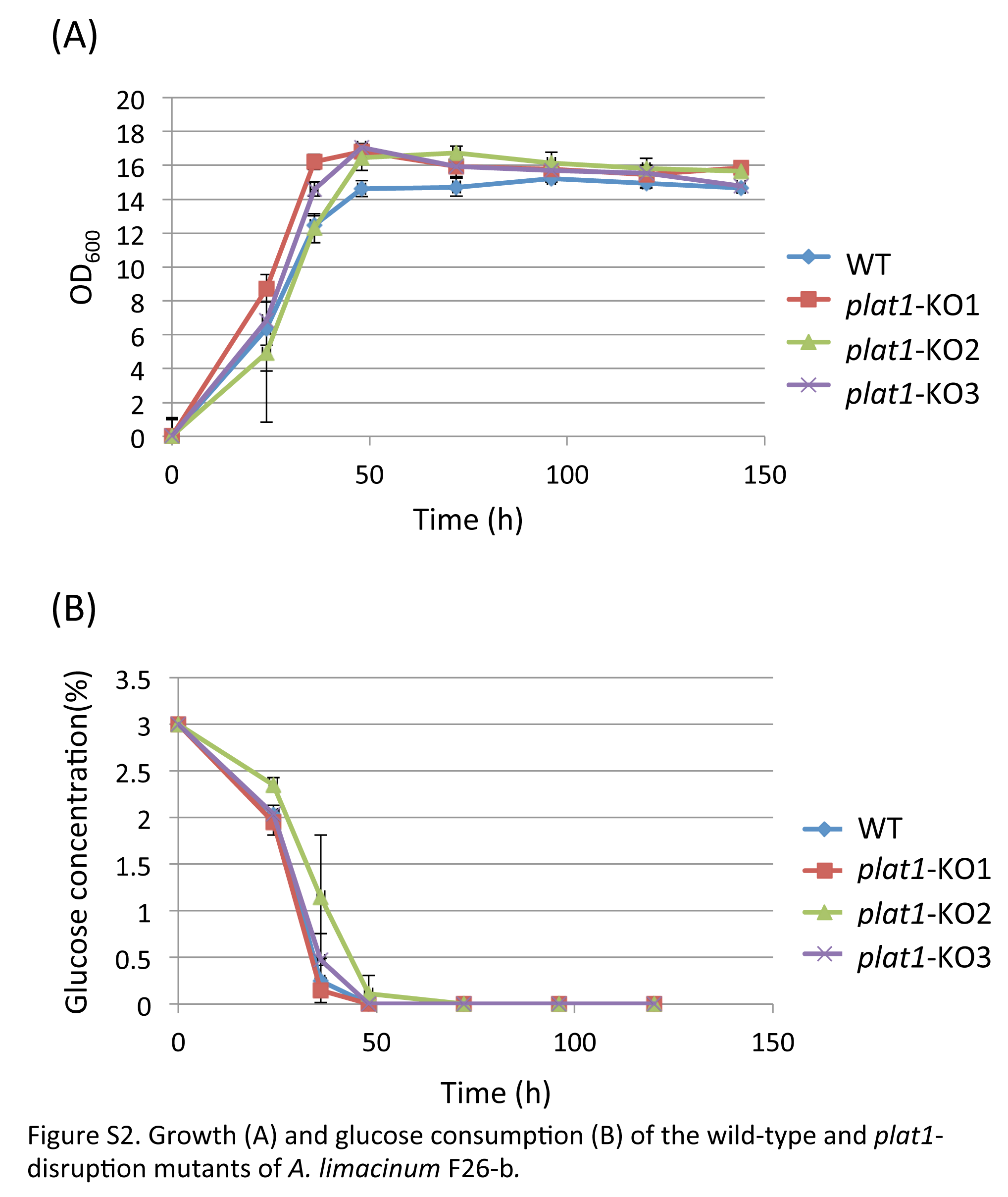

Supplement: Figure S2 — Growth (A) and glucose consumption (B) of the wild-type and plat1 -disruption mutants of A. limacinum F26-b. The wild-type and three plat1-disrupted mutants (plat1-KO1∼3) were cultured in 50-ml flasks containing 20 ml of GY medium (3% glucose and 1% yeast extract in 50% artificial sea water) containing 0.1% vitamin mixture (vitamin B1: 200 mg, vitamin B2: 1 mg, vitamin B12: 1 mg/100 ml distilled water) at 25°C for the periods indicated. A small sample of the culture was withdrawn and the optical density at 600 nm was measured after suitable dilution. The glucose content of the culture supernatant was measured with a glucose CII-test (Wako, Japan). Data represent the mean ± SD (n = 3). (TIF) [file pone.0102377.s002.tif]
