# Supplementary material for: Novel Lysophospholipid Acyltransferase PLAT1 of Aurantiochytrium limacinum F26-b Responsible for Generation of Palmitate-Docosahexaenoate-Phosphatidylcholine and Phosphatidylethanolamine
Source: PLoS One. 2014 Aug 4;9(8):e102377. doi: 10.1371/journal.pone.0102377 (PMC4121067; doi:10.1371/journal.pone.0102377)
Supplement: Table S1 — PCR primers used in this study. Underlines, restriction enzyme sites (No. 3 HindIII, No. 4 BamHI, No. 5 BglII, No. 6 SalI); Dotline, FLAG tag sequence. (DOCX) [file pone.0102377.s003.docx]

**Table S1**. PCR primers used in this study.

| Primer  　　No. | 5’ 3’ |
| --- | --- |
| 1 | TAATGGCCATGGGACGCGAGTC |
| 2 | AAACATGTTCAGTAATTTACTTCCCG |
| 3 | AGAAGCTTCATGGATTACAAGGATGACGATGACAAGGCCATGGGACGG |
| 4 | GGCGCGGATCCGCTTAATTTGATTTCTTAACCTTGGG |
| 5 | CGCAGATCTTTAGTTTCATCACGACCC |
| 6 | ATTGTCGACCCTCTCTAAACGCTTCCC |
| 7 | ATCTTGCTTTTGACCCGTCTTGTCC |
| 8 | CGAAGCTCAGGTCCTCTAGATAC |

Underlines, restriction enzyme sites (No.3 *Hin*dIII, No.4 *Bam*HI, No. 5 *Bgl*II, No. 6 *Sal*I); Dotline, FLAG tag sequence
