# Supplementary material for: Novel Lysophospholipid Acyltransferase PLAT1 of Aurantiochytrium limacinum F26-b Responsible for Generation of Palmitate-Docosahexaenoate-Phosphatidylcholine and Phosphatidylethanolamine
Source: PLoS One. 2014 Aug 4;9(8):e102377. doi: 10.1371/journal.pone.0102377 (PMC4121067; doi:10.1371/journal.pone.0102377)
Supplement: Table S2 — Ratios of PL species in wild-type and plat1-disrupted mutants. Values are the ratios of 16:0-DHA-PLs to DHA-DHA-PLs, calculated from the data of nanoESI/MS analyses. WT, wild-type of A.limacinum F26-b; KO1∼KO3, three different plat1-disrupted mutants obtained from transfection of the wild-type with a KO construct containing the HygR gene as a marker, as shown in Figure S1. (DOCX) [file pone.0102377.s004.docx]

**Table S2.** Ratios of PL species in wild-type and *plat1*-disrupted mutants.

| PLs (fatty acid species) | Strains | | | |
| --- | --- | --- | --- | --- |
|  | WT | KO1 | KO2 | KO3 |
|  | Ratio | | | |
| PC (16:0-DHA/DHA-DHA) | 3.43 | 2.22 | 2.69 | 2.32 |
| PE (16:0-DHA/DHA-DHA) | 4.06 | 3.76 | 3.32 | 3.55 |

Values are the ratios of 16:0-DHA-PLs to DHA-DHA-PLs, calculated from the data of nanoESI/MS analyses.

WT, wild-type of *A.limacinum* F26-b; KO1~KO3, three different *plat1*-disrupted mutants obtained from transfection of the wild-type with a KO construct containing the HygR gene as a marker, as shown in Figure S1.
